# The Poor Survival among Pulmonary Tuberculosis Patients in Chiapas, Mexico: The Case of Los Altos Region

**DOI:** 10.1155/2012/708423

**Published:** 2012-05-30

**Authors:** J. C. Nájera-Ortiz, H. J. Sánchez-Pérez, H. Ochoa-Díaz-López, G. Leal-Fernández, A. Navarro-Giné

**Affiliations:** ^1^Área Académica Sociedad, Cultura y Salud, El Colegio de la Frontera Sur (Ecosur), 29290 San Cristóbal de Las Casas, Chiapas, Mexico; ^2^Grups de Recerca d'Amèrica i Àfrica Latines, GRAAL-Ecosur, San Cristóbal de Las Casas, Chiapas, Mexico; ^3^Departamento de Atención a la Salud, Universidad Autónoma Metropolitana-Xochimilco, 04960 México, DF, Mexico; ^4^Grups de Recerca d'Amèrica i Àfrica Latines (GRAAL), Unitat de Bioestadística, Facultat de Medicina, Universitat Autónoma de Barcelona (UAB), 08193 Barcelona, Spain

## Abstract

*Objective*. To analyse survival in patients with pulmonary tuberculosis (PTB) and factors associated with such survival. *Design*. Study of a cohort of patients aged over 14 years diagnosed with PTB from January 1, 1998 to July 31, 2005. During 2004–2006 a home visit was made to each patient and, during 2008-2009, they were visited again. During these visits a follow-up interview was administered; when the patient had died, a verbal autopsy was conducted with family members. Statistical analysis consisted of survival tests, Kaplan-Meier log-rank test and Cox regression. *Results*. Of 305 studied patients, 68 had died due to PTB by the time of the first evaluation, 237 were followed-up for a second evaluation, and 10 of them had died of PTB. According to the Cox regression, age (over 45 years) and treatment duration (under six months) were associated with a poorer survival. When treatment duration was excluded, the association between poorer survival with age persisted, whereas with having been treated via DOTS strategy, was barely significant. *Conclusions*. In the studied area it is necessary that patients receive a complete treatment scheme, and to give priority to patients aged over 45 years.

## 1. Introduction

Tuberculosis (TB) continues to be one of the leading causes of disease, disability, and death. Seventy-five percent of people with TB belong to the economically active age group (15–54 years) and 95% of the cases and 99% of deaths occur in developing countries [1]. 

The incidence rate of TB in Mexico in 2007 was 20/100,000 and the death rate was 2.4/100,000 [2]. Nevertheless, given the socioeconomic conditions in the country such as high rurality, high levels of poverty, and shortage of health resources in marginalized areas, both figures might be higher.

Chiapas is among the Mexican states with the highest indices of poverty [3]. This state shows one of the highest proportions of rural and indigenous population, has the lowest human development index of the whole country [3], has the lowest *per capita* availability of health resources [4, 5], and has the highest rates of prevalence and mortality due to TB [6]. 

This situation is even worse in rural indigenous populations of Chiapas, like Los Altos region, where in many communities access to basic services turns out to be more a privilege than a right. In such circumstances, control and anti-TB treatment of TB patients via the directly observed treatment short-course (DOTS) strategy is very difficult. Therefore the results of this situation are high levels of under-diagnosis and of treatment defaulting, multidrug resistance, and deaths due to TB [7–9]. 

In Los Altos region of Chiapas, despite the well-known high underdiagnosis of cases [10], the recorded levels of morbimortality due to TB are very high. The poor living and health conditions of Chiapas together with a deficient population coverage and poor quality of the health care services and the high prevalence of diseases such as diabetes, HIV/AIDS, malnutrition, and alcoholism might be associated with the poor survival of TB patients [7]. 

The survival in patients with pulmonary tuberculosis (PTB) has not been analysed before in Chiapas. Therefore, the aim of the present study was to analyse survival among patients diagnosed with PTB during the period 1998–2005 in Los Altos region of Chiapas and to investigate socioeconomic, demographic, and anti-TB treatment-related factors associated with their survival.

## 2. Study Population and Methods

A longitudinal prospective study was carried out in a cohort of patients aged 15 years and over, diagnosed with PTB by the Health District of Los Altos Region (Ministry of Health) by acid fast smear or culture, between 1 January 1998 and 31 July 2005 in Los Altos region of Chiapas.

The study began in 2004-2005 with an initial phase which consisted of visiting the homes of those patients diagnosed between 1998 and 2002 (*n* = 431) [7]. Subsequently in 2006, a second visit was made to locate those patients diagnosed between 2003 and July 2005 (*n* = 98). All patients (*n* = 529) were evaluated in terms of their PTB status.

The disturbing findings of the first study (95 deaths—of which 68 were probably due to PTB—plus 237 located alive, and 197 not found) motivated a second study involving all patients located alive in the first study (*n* = 237). We undertook a follow-up visit to these patients during 2008-2009. Out of the 237 patients, 10 had died due to PTB, 175 were located alive, and 52 could not be located as they had moved.

In both studies, patients located alive were interviewed at home; when they had died, a verbal autopsy was carried out with their family, in which the date, place, and cause of death were investigated, among other aspects.

Survival time was defined as the number of days between the recorded date of PTB diagnosis and the date of death or, in the case of patients located alive, between diagnosis date and the date of their last interview.

Survival was analysed in terms of demographics (sex, age, type of community of residence, indigenous condition and whether they spoke Spanish or not), socioeconomic status (educational level, occupation, social security), treatment-related variables: treated under DOTS strategy or not, and duration of anti-TB treatment: complete or not (six months or less, resp.).

A patient treated under the DOTS strategy was defined as the one who is under the anti-TB drug intake program and he/she is directly and constantly supervised by either health staff or a trained relative.

The survival function was estimated using the Kaplan-Meier method [11], and survival curves were compared among different categories of a given variable using the Log-rank test [11]. Finally, for those variables found to be associated with survival time, two multivariate analyses (Cox regression) were carried out, one taking into account the duration of treatment and the other ignoring this duration due to the possibility of a correlation effect between duration of treatment and survival time, which could lead to overestimation of the true effect of treatment duration. For example, a patient who dies two months after he is diagnosed, might not have received more than two-month treatment.

In all statistical analyses a significance level of *α* = 0.05 was assumed. The analyses were conducted using the statistical packages *SPSS* version 15 [12] and Stata version 11.1 [13]. 

## 3. Results

### 3.1. Demographic and Socioeconomic Indicators of the Patients Studied

Table 1 shows the main demographic, socioeconomic, and treatment-related indicators for the 305 patients studied, according to their alive or deceased condition.

Only 12.8% of the patients studied reported having received their anti-TB treatment under DOTS strategy, and one in five (22.3%) had less than six months of anti-TB treatment.

### 3.2. Survival

The average follow-up time per patient for the entire cohort was 2,032 days (median 2,137; range 0 to 4,089), for patients located alive was 2,464 days (median 2,601; range 382 to 4,089), and for patients who died (from the PTB diagnosis date to their date of death) was 774 days (median 670; range 0 to 3,185). The incidence of mortality by person-years of follow-up was 4.6 by 100 person-years.

Of the 78 deaths suffering from PTB, 25% died during the six months following diagnosis (i.e., during treatment), 38% by the end of the first year from the date of diagnosis, 53% had died by the end of the second year, 72% after three years, 86% after four years, and 92.3% after five years; only one of the 78 patients who had died survived for seven years and over.

### 3.3. Factors Related to Survival

**Figure 1:**
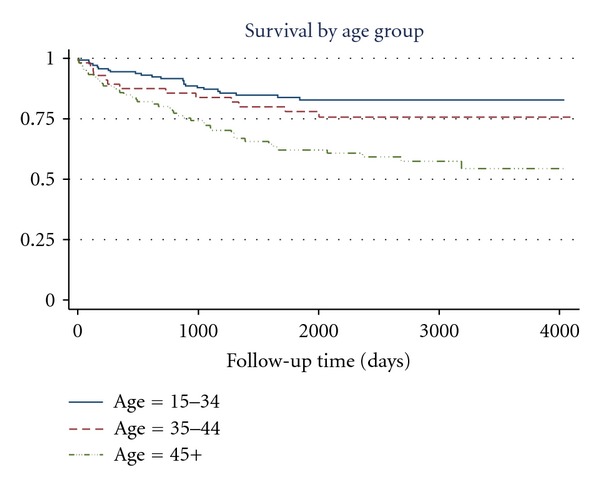
Survival functions for variable: Age.

**Figure 2:**
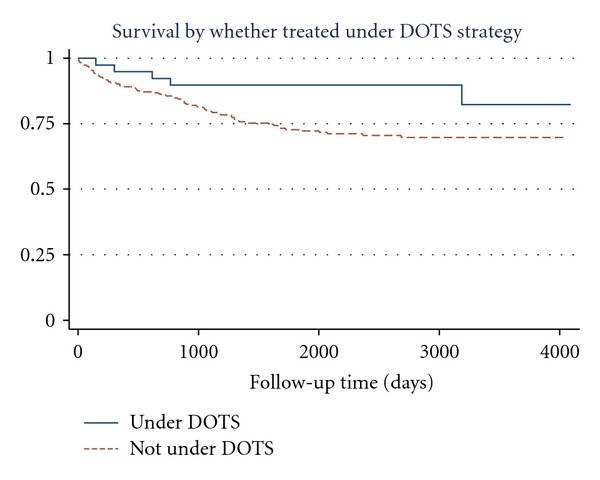
Survival functions for variable: treated under DOTS strategy.

**Figure 3:**
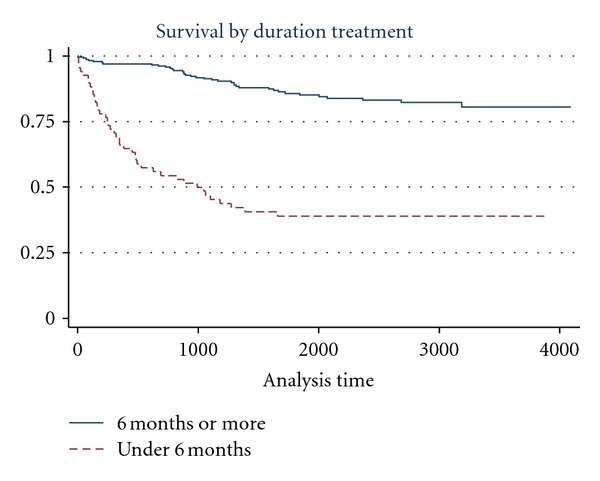
Survival functions for variable: duration of treatment.

No significant differences were found between survival curves in terms of the following variables: sex, indigenous condition, type of community of residence (rural-urban), occupation, or social security (yes or no). Statistically significant differences were observed for age (poorer survival in those aged over 45 years) (Figure 1), educational level (poorer survival in patients with under three years of schooling), whether treated via the DOTS strategy (poorer survival in those not treated under DOTS) (Figure 2), and duration of treatment (poorer survival in those not completing treatment) (Figure 3).

In the multivariate analysis performed, the only two variables which continued to show statistically significant associations with survival were age and the duration of anti-TB treatment. Patients aged 45 years and over (*n* = 106) presented poorer survival than the younger patients (Table 2). The median survival time of those in this age group who died (*n* = 42) was 718 days (range 0 to 3,185). Of these 42 only 9.5% received anti-TB treatment via the DOTS strategy, and in 47.6% the duration of such treatment was not completed. The median survival time of those who died in the reference group consisting of patients aged 15–34 years (23/143) was 688 days (range 8–1,841).

With regard to the duration of anti-TB treatment, those patients with incomplete treatment presented poorer survival than those treated with complete treatment (Table 2). The median survival time of the 41 patients out of those with incomplete treatment was 261 days (range 0–1,658), whereas among those dying in the other group (*n* = 37) the median survival time was 1,137 days (range 202–3,185).

## 4. Discussion

This study represents one of the first attempts to measure survival among PTB patients in Chiapas, Mexico, an area of high socioeconomic deprivation and strong presence of indigenous population.

From the cohort of PTB patients diagnosed between 1998 and 2005, and followed up between 2004 and 2008, 15% of them had died from PTB. Chiapas is one of the Mexican states with higher levels of PTB mortality. For example, in 2008 and 2009 the PTB mortality for the country was 1.8 and 1.7/100000, respectively, whereas in Chiapas the PTB mortality rate was 4.68 and 3.79 for the same years. Moreover, the studied region is one of the regions of Chiapas with the highest PTB morbidity and mortality rates. This is likely due to the fact that Los Altos region is one of the regions with the worst living conditions and highest level of poverty, with mainly rural population, a high proportion of indigenous communities, great problems of access to health services, and scarce opportunities to get a proper diagnosis [7]. 

Therefore, the results of this study are consistent with the situation described previously. The findings also provide evidence that in the study area, patients being aged 45 years and over, not having completed the established six months treatment, and not having been treated via the DOTS strategy are associated with an increased risk of dying from PTB.

The fact that patients aged 45 years and over have a lower chance of survival may be a reflection of the accumulation of unfavourable living conditions related to their poverty (lower education, higher levels of malnutrition during childhood, etc.) which probably made them more vulnerable to TB.

Regarding whether they completed six months of treatment or not, it should be mentioned that the study area is characterized by having a significant proportion of indigenous and peasant populations living in severe conditions of poverty and deprivation. Also, both access to and quality of health services are deficient. There is considerable evidence of the mistreatment of patients by the health services staff [14], deficient application of the DOTS strategy in anti-TB treatment (in the present study only 13% were treated under DOTS), and the frequent shortages of supply of anti-TB drugs [10]. All this contributes to have a high proportion of PTB patients who start anti-TB treatment but who do not complete it, leading to a higher mortality among these patients, as well as increasing their chances of becoming multidrug resistant cases [9]. 

Thus, the fact that the patients whose treatment duration was less than six months had a lower chance of survival is consistent with the documented evidence that points out that six-month treatment is the minimum time for a person to have a 96–98% chance of being cured (in new cases) [15]. Of those patients receiving anti-TB treatment of less than six-month duration (*n* = 68), 60% had died by the time of this study, whereas the proportion of deceased patients among those treated for six months or more was 15.6%.

The high proportion of deceased patients found by this study (26% of those patients with the required information to be included in the survival analyses) is much higher than that found in a similar study conducted in Peru [16], where only 4.5% had died. The differences in findings between these two studies may be the result from the following two aspects: in our study the median days of patient follow-up was 2,137 days, whereas in the Peruvian study it was 178 days. In the Peruvian study the main factor associated with survival time was HIV-TB coinfection; in our study no data were available to assess this association.

Regarding survival time, we would like to stress the following points.

1. One quarter of deaths occurred in the first six months following their PTB diagnosis (during the time they were supposedly in treatment). It is also remarkable that two years after diagnosis slightly more than half of the deaths had already occurred. These two facts suggest, on the one hand, problems with the quality of health care services provided, such as delay in the diagnosis (at the moment when the health services made a PTB diagnosis, the patient might have already had a severe health condition). On the other hand, the living and health conditions of the patients under study are highly precarious, making them particularly vulnerable to PTB. However, it is noteworthy that another important proportion of deaths occurred between three and four years after diagnosis.
2. According to the literature reviewed [17], mortality among nontreated PTB patients is 50% at five years after diagnosis. In the present study, in which all patients were diagnosed and received anti-TB treatment (according to current Mexican legislation, all patients diagnosed with PTB must be treated under DOTS), mortality was 23.6% at five years following diagnosis. The importance of our results lies in the fact that the patients were diagnosed and consequently received anti-TB treatment. Thus, a necessary question arises: what survival can be expected among patients not diagnosed or treated? This is an important issue to consider given that the socioeconomic conditions and degree of deprivation in the region are mostly unfavourable. Several studies conducted in the communities of Chiapas have shown the high levels of underdiagnosis, reaching 55–76% of prevalent PTB cases in rural areas [18].

Regarding the study constraints, the following issues are pointed out.

- First is the high attrition rate of follow-up patients. We think that this situation is due to population movements. The migration in this region is mainly due to economic factors (patients by themselves or accompanying a relative look for better job opportunities; some rural patients move to the urban areas looking for health care). These patients are registered by the health services as urban patients when in fact they are not. Once they finished their treatment, they go back to their rural communities or look for another place to live without notifying it to the health services. Therefore, it is not possible to follow up them after they have finished their treatment.
- Among the group of 78 patients who died from PTB, only five were treated via DOTS. This did not make possible to undertake a multivariate analysis including the variable treatment via DOTS. The difference in the death proportion between those not treated via DOTS and those treated via DOTS was considerable (93.6% versus 6.4%, resp.).

## 5. Conclusions

The poor survival associated with age (45 years and over), with not having anti-TB treatment for six months, and not being treated via the DOTS strategy, may be considered as failures in the performance of the TB prevention and control programme in the study region. Thus, in the studied area, it is necessary to establish mechanisms to ensure that patients receive at least six months of anti-TB treatment and to pay special attention to patients aged 45 years and over.

## Figures and Tables

**Table 1:** Demographic, socioeconomic, and antituberculosis treatment-related indicators among the study patients.

Indicator (%)	Total *n* = 305	Alive *n* = 227	Deceased *n* = 78	Significance (*x* ^2^ Mantel-Cox)
Demographic				

Sex				0.556
Male	51.1	52.6	50.7	
Female	48.9	47.4	49.3	

Age group (years)				<0.001
15–34	46.9	52.9	29.5	
35–44	18.4	18.9	16.7	
45 and over	34.8	28.2	53.8	

Type of community of residence				0.493
Rural	69.8	69.2	71.8	
Urban	30.2	30.8	28.2	

Speak Spanish				0.328
Yes	69.2	70.5	65.4	
No	30.8	29.5	34.6	

Indigenous				0.903
Yes	85.6	85.9	84.6	
No	14.4	14.1	15.4	

Socioeconomic				

Educational level				0.020
0–3 years of schooling	73.1	69.2	84.6	
More than 3 years of schooling	26.9	30.8	15.4	

Occupation				0.113
Agricultural	41.6	39.2	48.7	
Non-agricultural	58.4	60.8	51.3	

With social security				0.102
Yes	22.0	22.9	19.2	
No	78.0	77.1	80.8	

Antituberculosis treatment related				

DOTS strategy treatment				0.041
Yes	12.8	15.0	6.4	
No	87.2	85.0	93.6	

Duration of treatment (completion)				<0.001
Not	22.3	11.9	52.6	
Yes	77.7	88.1	47.4	

**Table 2:** Deaths, incidence density, and hazard ratios according cox regression models.

Including variable “duration of treatment”^a^						
Factor	Number of deaths	Incidence density*	HR	95% CI		*P*

Age						
15–34 (*n* = 143)	23	2.8	1			
35–44 (*n* = 56)	13	3.7	1.03	0.51	2.10	0.927
45 and over (*n* = 106)	42	8.1	2.10	1.22	3.61	0.007

Duration of treatment (completion)						
Yes (*n* = 237)	37	2.6	1			
Not (*n* = 68)	41	16.0	5.74	3.59	9.18	<0.001

*Expressed in terms of 100 person-years of follow-up.

^a^The following variables were not statistically significant: sex (*P* = 0.234), educational level (*P* = 0.172), and DOTS treament (*P* = 0.145).
